# The development and validation of screening tools for semi-automated surveillance of surgical site infection following various surgeries

**DOI:** 10.3389/fmed.2023.1023385

**Published:** 2023-01-26

**Authors:** Pnina Shitrit, Michal Y. Chowers, Khitam Muhsen

**Affiliations:** ^1^Infection Control Unit, Meir Medical Center, Kfar Saba, Israel; ^2^Department of Epidemiology and Preventive Medicine, School of Public Health, Sackler Faculty of Medicine, Tel Aviv University, Tel Aviv, Israel; ^3^Infectious Disease Unit, Meir Medical Center, Kfar Saba, Israel; ^4^Sackler Faculty of Medicine, Tel Aviv University, Tel Aviv, Israel

**Keywords:** surgical site infection, semi-automated surveillance, screening tool, validation, model development

## Abstract

**Background:**

Surveillance of surgical site infections (SSIs) is essential for better prevention. We developed a screening method for SSIs in adults.

**Methods:**

The training dataset included data from patients who underwent orthopedic surgeries (*N* = 1,090), colorectal surgeries (*N* = 817), and abdominal hysterectomies (*N* = 523) during 2015–2018. The gold standard for the validation of the screening tool was the presence of SSI as determined by a trained infection control practitioner, *via* manual full medical record review, using the US Center for Disease Control and Prevention criteria. Using multivariable regression models, we identified the correlates of SSI. Patients who had at least one of these correlates were classified as likely to having SSI and those who did not have any of the correlates were classified as unlikely to have SSI. We calculated the sensitivity and specificity of this tool compared to the gold standard and applied the tool to a validation dataset (*N* = 1,310, years 2019–2020).

**Results:**

SSI was diagnosed by an infection control specialist in 8.2, 5.2, and 31.2% of the patients in the training dataset who underwent hysterectomies, orthopedic surgeries and colorectal surgeries, respectively, vs. 6.2, 6.6, and 25.5%, respectively, in the validation dataset. The correlates of SSI after abdominal hysterectomy were prolonged hospitalization, ordering wound or blood culture, emergency room visit and reoperation; in orthopedic surgery, emergency room visit, wound culture, reoperation, and documentation of SSI, and in colorectal surgeries prolonged hospitalization, readmission, and ordering wound or blood cultures. Area under the curve was >90%. The sensitivity and specificity (95% CI) of the screening tool were 98% (88–100) and 58% (53–62), for abdominal hysterectomy, 91% (81–96) and 82% (80–84) in orthopedic surgeries and 96% (90–98) and 62% (58–66) in colorectal surgeries. The corresponding values for the validation dataset were 89% (67–97) and 75% (69–80) in abdominal hysterectomy; 85% (72–93) and 83% (80–86) in orthopedic surgeries and 98% (93–99) and 59% (53–64) in colorectal surgeries. The number of files needed to be fully reviewed declined by 61–66.

**Conclusion:**

The presented semi–automated simple screening tool for SSI surveillance had good sensitivity and specificity and it has great potential of reducing workload and improving SSI surveillance.

## Introduction

Surgical site infection (SSI) is a main complication following surgery, which accounts for more than 20% of hospital-acquired infections (HAI) worldwide (1–4). The incidence of SSI might exceed 20%, depends on the type of surgery (5, 6). SSI is associated with longer hospital stay and higher mortality rates and poses a substantial burden to the healthcare system (1, 4).

Continuous surveillance of SSI coupled with periodic feedback to surgeons is essential for the prevention and control of SSI and was shown to be effective in reducing SSI in a variety of surgical categories such as colorectal and hepatobiliary surgeries (5–9).

Surveillance of SSI is complicated and requires data collection from multiple sources to accurately define the presence of the infection and determine both the numerator and denominator (10). Manual surveillance is time-consuming and labor intensive, and its accuracy relies on the quality and training of infection control practitioners. Moreover, the identification of post-discharge SSIs is challenging (11–14). With the development of electronic medical records (EMR), automated or semi-automated surveillance systems emerged for the monitoring and identification of HAIs, including SSIs (10–13, 15) showing effectiveness of such systems in reducing time and workload from infection control teams compared to manual surveillance (16–19). Despite this evidence, questions remain regarding the prediction ability of different patient or surgery characteristics, the combination of characteristics and whether it is possible to use one prediction model for all surgeries or there is need for customized models for different surgeries. Another unresolved issue is whether different models can be used with the same effectiveness in predicting SSI, allowing hospitals to develop local predicting models based on available data and not give up semiautomatic surveillance just because some variables cannot be retrieved by the hospital systems. Some gaps arising from current evidence include small sample size, a limited number of variables that were tested such as positive wound cultures (17), using variables that are specific for only some health systems such as infectious disease consultation (20), concentrating on clean surgeries with low SSI rates (21, 22) or using advanced clinical support systems which are not widely available (23).

The aim of the current study was to develop and validate a semi-automated screening tool for surveillance of SSIs in adults following various surgeries, namely abdominal hysterectomy, colorectal surgeries, spinal fusion, and joint replacements, using comprehensive yet simple demographic and clinical characteristics of the patient, surgical producers and management. Our underlying assumption was that some clinical and microbiological characteristics that can be easily obtained *via* collective reports might serve a good screening tool for SSIs. The rationale for the development of such a screening tool is to capture SSI events without the need to review medical records of all patients undergoing surgery, thus reducing the burden and workload on infection control teams.

## Materials and methods

### Study design and population

A historical cohort study was conducted among adults who underwent an abdominal hysterectomy, colorectal surgeries, spinal fusion, and joint replacements at Meir Hospital during 2015–2020.

Meir Medical Center is a secondary 780-bed university-affiliated hospital. It serves a population of about 600,000 individuals, with about 20,000 surgeries being conducted annually.

Manual surveillance of SSIs was ongoing in this center since 2008 for selected surgeries. Surveillance was carried out for in-hospital and post-discharge SSI using the data from patient’s medical records, return visits to the emergency room, readmission, reoperation, outpatient clinic visits, and a telephone interview with the patient 30 days after surgery. The ability to expand the surveillance to multiple surgery categories was compromised due to the substantial time and workload required for manual chart review. In 2014, an EMR system was introduced into the hospital’s systems, which enabled the extraction of computerized reports, including demographics and clinical data. In a pilot study, we validated a semi-automated surveillance system for SSI following a cesarean section using a dynamic report obtained from the EMR. Compared to manual surveillance, the surveillance based on the electronic report, significantly reduced the number of charts needed for review and reached a sensitivity and specificity of above 95% (19). These results were the basis of the current study.

### Data collection and definition of the study variables

We utilized data from a cohort of patients who underwent an abdominal hysterectomy, colorectal surgeries, spinal fusion, and joint replacements, and were included in a local SSI manual surveillance program of our center. A trained infection control practitioner manually reviewed the medical records of all patients. Information was collected on the following independent variables: age (in years), sex, diagnosis of diabetes, receiving antibiotic prophylaxis before surgery, operation duration (in hours), ordering blood culture, ordering wound culture, the National Nosocomial Infection Surveillance (NNIS) risk index that consists of operation length, wound class (i.e., clean, clean contaminated, contaminated, or dirty), and the American Society of Anesthesiologists (ASA) score, length of hospital stay before surgery and from surgery to discharge (in days). The variable length of stay (LOS) from surgery to discharge was categorized using the 75th percentile of patients without SSI to define prolonged hospitalization, and it was specific for each surgery category.

Moreover, we collected data on returning to the emergency room of our hospital within 30–90 days, readmission or reoperation 30 or 90 days after surgery and recording SSI diagnosis in the medical record by the attending physicians.

The main dependent variable was the presence of SSI as determined by the infection control practitioner (yes or no). We collected information on the type of SSI: superficial, deep, or organ/space. SSI was defined using the using the US Center for Disease Control and Prevention criteria (24). The main outcome variable was SSI within 30 days from surgery for abdominal hysterectomies, colorectal surgeries, and superficial SSI in orthopedic surgeries, or 90 days after surgery or deep/organ space infection in joint replacement/spinal fusion. We included infections that were identified during hospitalization and after discharge from hospital.

### Statistical methods

Data analysis was performed separately for each surgery category: abdominal hysterectomy; orthopedic surgery (joint replacement/spinal fusion) and colorectal surgery.

Data from the years 2015–2018 were used as the training dataset and the data from 2019 to 2020 were used as the validation dataset. We compared patient and surgery characteristics between the two datasets using the Student’s *t*-test for continuous variables or the Mann–Whitney test for variables that did not follow a normal distribution, and the chi square or Fisher exact where appropriate for categorical variables.

Missing data was low (0.8–1% in only 3 variables) therefore we preformed complete case analysis only.

#### Development of the screening tool for SSI surveillance

Initially we identified factors that were associated with the development of SSI. Using the training dataset, we compared demographic and clinical characteristics of patients who developed SSI with those who did not develop SSI using the chi square or Fisher’s exact test for categorical variables and Student’s *t* test for continuous variables. The Mann Whitney test was used for variables with skewed distribution. Multivariable logistic regression models were performed, in which SSI was the dependent variable. Independent variables that were associated with SSI in the bivariate analysis with a *p* < 0.2 were considered in the multivariable model. The selection of the final model was made using the area under the receiver operating characteristic [ROC] curve and calibration measure (Hosmer and Lemeshow test). The ROC curves were calculated using the predicted probability from the logistic regression model against the diagnosis of SSI by an infection control physician.

Next we used the independent variables that were identified in the multivariable models in developing a screening tool for SSI. The screening tool comprised a new variable that we created, in which patients who had at least one of the independent variables that were included in the multivariable model were considered as likely having SSI, while those who did not have any of these variables were considered as unlikely having SSI. We compared the classification of patients using this screening tool of SSI (likely or unlikely having SSI, a dichotomous variable), with the diagnosis of SSI by an infection control physician, which was used as the gold standard. We calculated the sensitivity, specificity, negative predictive value (NPV) and positive predictive value (PPV) and the corresponding 95% confidence intervals (CIs) of the screening tool compared to the physician’s diagnosis of SSI. The rationale of applying this strategy was to achieve maximum sensitivity, while reducing the number of medical records that should be to be fully reviewed by the infection control team in monitoring SSIs.

#### Validation of the screening tool of SSI

Validation of the SSI screening tool that was developed in the initial step was undertaken using the validation dataset. We fitted a logistic regression model in which SSI was the dependent variable, while the independent variables were those that we identified in the previous step of developing the screening tool. We calculated ROC curves, sensitivity, specificity, PPV and NPV as described above.

The data were analyzed using SPSS version 27 (IBM, Armonk, New York, NY, USA) and WINPEPI version 11.65 (25).

### Ethical considerations

The data used in this study were collected for purposes of infection control surveillance as part of improvement program. This was a retrospective study using medical records. The study protocol was approved by the Institutional Review Board Committee of Meir Medical Center and an exemption from signing an informed consent was given, because of the retrospective study design.

## Results

### Description of the study sample

Data of 3,744 surgeries performed in 3,620 patients were analyzed; of these, 2,434 surgeries (2,341 patients) were conducted during 2015–2018 and included in the training dataset and 1,310 surgeries (1,279 patients) were conducted during 2019–2020 and were included in the validation dataset.

The training dataset (years 2015–2018) included 523 abdominal hysterectomies, 1,090 orthopedic surgeries and 817 colorectal surgeries. The respective patient’s median age in these surgeries was 55.0, 67.2, and 69.1 years. Overall 15.6% of the orthopedic surgeries and 35.0% of the colorectal surgeries were urgent surgeries, compared to 2.5% of the abdominal hysterectomies. General anesthesia was used in all women who underwent hysterectomy, in 87.3 and 96.1% of the patients who underwent orthopedic and colorectal surgeries, respectively. Most patients (64.5–82.9%) had low risk index (defined as NNIS risk index 0–1 scores) (Table 1).

**TABLE 1:** Baseline characteristics of patients included in the training and validation datasets by surgery category.

	Abdominal hysterectomy			Orthopedic surgery (joint replacement/spinal fusion)			Colorectal surgery		
	Training dataset (2015–2018)	Validation dataset (2019–2020)	*P*	Training dataset (2015–2018)	Validation dataset (2019–2020)	*P*	Training dataset (2015–2018) (%)	Validation dataset (2019–2020)	*P*
N	523	289		1,090	609		817	412	
Age, years, median (IQR)	55.0 (46.7–66.7)	55.0 (47–68.5)	0.38	67.2 (58.2–73.9)	68.0 (60.0–75.0)	0.22	69.1	67.5	0.06
Sex (male), N (%)	N/A	N/A		477 (43.8)	294 (48.3)	0.07	403 (49.3)	208 (50.5)	0.7
Urgent surgery, N (%)	13 (2.5)	4 (1.4)	0.29	170 (15.6)	171 (28.1)	<0.001	286 (35.0)	143 (34.7)	0.92
Anesthesia (general), N (%)	523 (100.0)	285 (98.6)	0.16	952 (87.3)	474 (77.8)	<0.001	785 (96.1)	410 (99.1)	<0.01
Operation duration, hours, median (25th–75th percentiles)	2.5 (2.1–3.4)	2.3 (2.1–3.1)	0.004	2.2 (1.6–3.0)	1.6 (1.4–2.3)	<0.001	3.0 (2.1–4.0)	2.5 (2–3.5)	0.008
LOS from surgery to discharge, days, median (25th–75th percentiles)	3 (2–4.3)	2 (2–4)	<0.001	5.0 (4–7)	4 (3–7)	<0.001	7 (5–11.5)	6 (4–10)	0.015
Prolonged hospitalization*	158 (30.2)	40 (13.8)	<0.001	239 (21.9)	140 (23.0)	0.61	301 (36.8)	142 (34.5)	0.41
Risk index, N (%)			0.26			0.005			0.07
0	180 (34.6)	84 (29.5)		557 (51.3)	253 (42.4)		212 (26.2)	85 (20.6)	
1	251 (48.3)	143 (50.2)		398 (36.7)	262 (43.9)		387 (47.8)	196 (47.6)	
2–3	89 (17.1)	58 (20.4)		130 (23.3)	82 (32.4)		210 (25.9)	131 (31.8)	
Wound class, N (%)			0.77			<0.001			0.082
Clean	NA	NA		1029 (94.4)	566 (92.9)		NA	NA	
Clean-contaminated	517 (98.9)	285 (98.6)		35 (3.2)	5 (0.8)		645 (78.9)	303 (73.5)	
Contaminated	3 (0.6)	1 (0.3)		2 (0.2)	4 (0.7)		57 (7.0)	32 (7.8)	
Dirty	3 (0.6)	3 (1.0)		24 (2.2)	34 (5.6)		115 (14.1)	77 (18.7)	
Received antibiotic prophylaxis, N (%)	504 (97.9)	283 (97.9)	0.95	1054 (98.8)	592 (97.2)	0.02	722 (89.2)	382 (93.7)	0.05
Surgical site infection (SSI), N (%)	43 (8.2)	18 (6.2)	0.3	57 (5.2)	40 (6.6)	0.25	255 (31.2)	105 (25.5)	0.04
Post-discharge SSI, N (% of SSI)	27 (62.8)	13 (72.2)	0.48	44 (75.9)	38 (82.6)	0.40	53 (20.7)	24 (21.1)	0.94
Infection type, N (% of SSI)			0.067			0.58			0.20
Superficial	13 (30.2)	11 (61.1)		25 (41.7)	14 (32.6)		106 (41.2)	51 (47.7)	
Deep	3 (7)	1 (5.6)		20 (33.3)	15 (34.9)		62 (24.1)	17 (15.9)	
Organ/space	27 (62.8)	6 (33.3)		15 (25)	14 (32.6)		89 (34.6)	39 (36.4)	

LOS, length of stay; N/A, not applicable; SSI, surgical site infection.

*Prolonged hospitalization was defined as length of stay of more than 4 days, for women who underwent abdominal hysterectomy, more than 7 days in orthopedic surgeries and more than 8 days for patients who underwent colorectal surgery. These cutoffs were determined using the 75th percentiles of length of stay among patients who did not have SSI.

*P*-value was obtained by the Student’s *t*-test for continuous variables or the Mann–Whitney test for variables that did not follow a normal distribution, and the chi square or Fisher exact where appropriate for categorical variables. Data on ASA score were missing for 14 patients in the training dataset and 16 patients in the validation dataset, for the risk index data were missing for 16 patients in the training and validation dataset each, and for antibiotic prophylaxis for data were missing for 39 patients in the training dataset only.

The validation dataset (years 2019–2020) included 289, 609, and 412 patients who underwent abdominal hysterectomy, orthopedic surgery, and colorectal surgery, respectively. The baseline characteristics of patients who underwent abdominal hysterectomy in the training and validation datasets were comparable, except of longer hospitalization (median of 3 vs. 2 days) and longer duration operation (median of 2.5 vs. 2.3 h) in the training dataset. The validation and training datasets of patients who underwent orthopedic surgeries differed significantly in the proportion of using general anesthesia, urgent surgeries, operation duration, hospitalization duration, NNIS risk index, and wound class. Patients who underwent colorectal surgeries in the training dataset differed significantly compared with those in the validation dataset in those who had general anesthesia (96.1 vs. 99.1%), operation duration (median of 3.0 vs. 2.5 h), hospitalization duration (median of 7 vs. 6 days) and the proportion of those who received prophylactic antibiotic therapy (89.2 vs. 93.7%) (Table 1).

The training dataset included 43 (8.2%), 57 (5.2%), and 255 (31.2%) patients who developed SSI among those who underwent hysterectomy, orthopedic surgery and colorectal surgery, respectively, compared to 18 (6.2%), 40 (6.6%), and 105 (25.5%) in the validation dataset (Table 1).

### Factors associated with SSI—Training dataset

A higher proportion of males was found among patients who underwent colorectal surgery and developed SSI than those without SSI (56.5 vs. 46.1% *p* = 0.006), but such difference was not found among patients who underwent orthopedic surgeries. Urgent surgery was more common among patients who underwent colorectal surgery and developed SSI than those who did not (46.8 vs. 28.8% *p* < 0.001), and the proportion of patients who received prophylaxis was lower in the SSI group (82.0 vs. 92.5%, *p* < 0.001). Such differences were not significant in the abdominal hysterectomy and orthopedic surgeries. Emergency room visits, readmission, ordering blood culture and wound culture, prolonged LOS, reoperation, and documentation of SSI in the medical chart were significantly more common among patients who developed SSIs than those who did not in all surgery categories. The proportion of patients who had high NNIS risk index was significantly higher among patients with SSI compared to those without SSI in the orthopedic (11.2 vs. 26.3% *p* < 0.001) and colorectal (22.0 vs. 34.8% *p* < 0.001) surgeries but not in abdominal hysterectomy. High-grade wound (contaminated/dirty) was more common in the SSI group vs. no SSI group in the colorectal surgeries only (32.2 vs. 16.0%, *p* < 0.001) (Table 2).

**TABLE 2:** Factors associated with surgical site infection by surgery category; bivariate analysis by surgery category—training dataset.

	Abdominal hysterectomy (*N* = 523)			Orthopedic surgery (*N* = 1,090)			Colorectal surgery (*N* = 817)		
	No SSI (*N* = 480)	SSI (*N* = 43)	*P*	No SSI (*N* = 1034)	SSI (*N* = 56)	*P*	No SSI (*N* = 562)	SSI (*N* = 255)	*P*
Age, years median (25th–75th percentiles)	55.5 (46.8–67.2)	50.7 (46.2–61.5)	0.07	67.2 (58.2–73.9)	68.1 (59.1–73.1)	0.7	69.3 (58.4–78.5)	68.5 (55.0–78.8)	0.4
Sex, male, N (%)	NA	NA		456 (44.1)	21 (36.8)	0.28	259 (46.1)	144 (56.5)	0.006
Anesthesia (general), N (%)	480 (100.0)	43 (100.0)		899 (87.0)	53 (93.0)	0.2	543 (96.6)	242 (94.9)	0.24
Urgent surgery, N (%)	9 (1.9)	4 (9.3)	0.16	157 (15.2)	13 (22.8)	0.12	162 (28.8)	124 (46.8)	<0.001
Received antibiotic prophylaxis, N (%)	464 (98.1)	40 (95.2)	0.22	998 (98.7)	56 (100.0)	0.4	517 (92.5)	205 (82.0)	<0.001
Emergency room visit, N (%)	76 (15.8)	20 (46.5)	<0.001	97 (9.4)	30 (52.6)	<0.001	48 (8.5)	43 (16.9)	<0.001
Readmission, N (%)	37 (7.7)	19 (44.2)	<0.001	156 (15.1)	43 (75.4)	<0.001	76 (13.5)	81 (31.8)	<0.001
Blood culture was ordered, N (%)	24 (5.0)	24 (55.8)	<0.001	103 (10.0)	35 (61.4)	<0.001	70 (12.5)	146 (57.3)	<0.001
Wound culture was ordered, N (%)	11 (2.3)	20 (46.5)	<0.001	68 (6.6)	48 (84.2)	<0.001	37 (6.6)	195 (76.5)	<0.001
Reoperation, N (%)	22 (4.6)	17 (39.5)	<0.001	67 (6.5)	38 (66.7)	<0.001	71 (12.6)	102 (40.0)	<0.001
Documentation of SSI diagnosis in the medical file, N (%)	1 (0.2)	4 (9.3)	<0.001	5 (0.5)	17 (29.8)	<0.001	2 (0.4)	4 (1.6)	0.08
High-grade ASA (>3), N (%)	127 (26.5)	12 (27.9)	0.84	300 (29.0)	27 (47.4)	0.003	201 (35.8)	119 (46.7)	0.003
High-risk index (2–3), N (%)	78 (16.4)	11 (25.6)	0.12	115 (11.2)	15 (26.3)	<0.001	123 (22)	88 (34.8)	<0.001
High-grade wound class (contaminated, dirty), N (%)	4 (0.8)	2 (4.7)	0.08	26 (2.5)	0 (0.0)	0.4	90 (16.0)	82 (32.2)	<0.001
Diabetes, N (%)	52 (10.8)	2 (4.7)	0.3	242 (23.4)	17 (29.8)	0.27	86 (15.3)	49 (19.2)	0.16
Operation duration, hours, median (IQR)	2.54 (2.07–3.35)	3.17 (2.08–6.0)	0.068	2.20 (1.58–2.58)	2.35 (2.05–3.20)	0.01	3.0 (2.1–4.0)	3.0 (2.2–4.1)	0.3
LOS before operation, days, median (IQR)	1 (0.84–1)	1 (0.84–1)	0.4	1.0 (0.82–1)	1.0 (0.95–2.0)	0.03	1 (0.9–1.0)	1 (0.9–1.3)	0.5
LOS from surgery to discharge, days, median (IQR)	3.0 (2–4.1)	5 (3–11)	<0.001	5 (3.5–6.5)	6.6 (5.0–11.8)	<0.001	6 (4.1–8.0)	12 (7.2–20.3)	<0.001
Prolonged hospitalization, N (%)*	131 (27.3)	27 (62.8)	<0.001	288 (27.9)	33 (57.9)	<0.001	125 (22.2)	176 (69.0)	<0.001

*Prolonged hospitalization was defined as length of stay of more than 4 days, for women who underwent abdominal hysterectomy, more than 7 days in orthopedic surgeries and more than 8 days for patients who underwent colorectal surgery. These cutoffs were determined using the 75th percentiles of length of stay among patients who did not have SSI.

ASA, American Society of Anesthesiologists; LOS, length of stay; SSI, surgical site infection.

*P*-value was obtained by the Student’s *t*-test for continuous variables or the Mann–Whitney test for variables that did not follow a normal distribution, and the chi square or Fisher exact where appropriate for categorical variables.

A multivariable model showed that emergency room visit, having blood culture or wound culture ordered, prolonged hospitalization (more than 4 days), and reoperation were significantly associated with SSI likelihood among women who underwent abdominal hysterectomy. Another analysis of patients who underwent orthopedic surgeries showed that emergency room visit, having a wound culture ordered, reoperation, and having documentation of SSI in the medical record were significantly associated with SSI likelihood. The variables that were significantly correlated with SSI in patients who underwent colorectal surgery (overall) were emergency room visits, having a wound or blood culture ordered, prolonged hospitalization (more than 8 days) and readmission. Limiting the analysis to elective colorectal surgeries, showed that ordering a wound culture, readmission and prolonged hospitalization were the significant variables (Table 3). Similar results were found when analyzing separately patients with clean contaminated wound class.

**TABLE 3:** Multivariable logistic regression models of factors associated with the development of SSI by surgery category—training dataset^a^.

Surgery category	Variable	Adjusted OR	95% CI	*P*-value	Negelkerke *R* square	AUC (95% CI)^c^
Abdominal hysterectomy^b^					0.42	0.90 (0.86–0.95)
	LOS > 4 days (vs. 4 days or less)^a^	3.4	1.5–8.0	0.004		
	Emergency room visit (yes vs. no)	4.0	1.7–9.4	0.002		
	Wound culture ordered (yes vs. no)	4.2	1.3–14.0	0.02		
	Blood culture ordered (yes vs. no)	4.9	1.8–13.0	0.001		
	Reoperation (yes vs. no)	2.9	1.0–8.7	0.051		
Orthopedic surgery^b^					0.58	0.93 (0.89–0.98)
	Emergency room visit (yes vs. no)	5.8	2.6–12.7	<0.001		
	Wound culture ordered (yes vs. no)	23.6	9.2–60.6	<0.001		
	Reoperation (yes vs. no)	3.2	1.4–7.8	0.008		
	Documentation of SSI diagnosis in the medical chart (yes vs. no)	9.3	2.5–35.3	0.001		
Colorectal surgery, all patients^b^					0.63	0.92 (0.89–0.94)
	Emergency room visit (yes vs. no)	2.1	1.09–4.2	0.027		
	Wound culture ordered (yes vs. no)	26.8	16.5–43.6	<0.001		
	Blood culture ordered (yes vs. no)	1.6	1.0–2.8	0.045		
	Readmission (yes vs. no)	1.9	1.1–3.4	0.017		
	LOS > 8 days (vs. 8 days or less)^a^	3.6	2.296–5.8	<0.001		
Elective colorectal surgery^b^					0.62	0.91 (0.88–0.95)
	Readmission (yes vs. no)	4.0	2.0–8.1	<0.001		
	LOS > 8 days (vs. 8 days or less)^a^	4.0	2.2–7.2	<0.001		
	Wound culture ordered (yes vs. no)	42.4	22.3–80.6	<0.001		
Urgent colorectal surgery^b^					0.60	0.90 (0.87–0.94)
	Emergency room visit (yes vs. no)	2.5	0.9–7.0	0.086		
	Wound culture ordered (yes vs. no)	16.9	8.4–33.7	<0.001		
	Blood culture ordered (yes vs. no)	2.0	1.0–4.0	0.065		
	Reoperation (yes vs. no)	2.4	1.0–5.8	0.050		
	LOS > 8 days (vs. 8 days or less)^a^	3.2	1.6–6.5	0.001		
Clean-contaminated colorectal surgery^b^					0.60	0.90 (0.88–0.93)
	Wound culture ordered (yes vs. no)	30.7	17.7–53.2	<0.001		
	LOS > 8 days (vs. 8 days or less)^a^	5.1	3.0–8.6	<0.001		
	Emergency room visit (yes vs. no)	2.7	1.3–5.8	0.01		
	Readmission (yes vs. no)	2.3	1.1-4.3	0.014		

^a^AUC, area under the curve; CI, confidence intervals; LOS, length of stay; OR, odds ratio; SSI, surgical site infection. Prolonged hospitalization was defined as a length of stay of more than 4 days, for women who underwent abdominal hysterectomy and more than 8 days for patients who underwent colorectal surgery. These cutoffs were determined using the 75th percentile of the length of stay among patients who did not have SSI.

^b^*P*-value by Hosmer and Lemeshow was 0.31, 0.90, 0.09 for abdominal hysterectomy, orthopedic surgeries, and colorectal surgeries (all patients). The respective *P*-values for elective colorectal surgeries, clean contaminated wound class, and urgent colorectal surgery were 0.07, 0.24, and 0.03.

^c^Area under the curve of the entire model.

A multivariable model that included only patients who underwent an urgent colorectal surgery showed that ordering wound or blood culture, prolonged hospitalizations, emergency room visit and reoperation that the significant correlates of SSI. The AUC was >90% in all these models (Table 3).

### Validity of the screening tool

Using the results of the multivariable models on the correlates of SSI (Table 3), we classified patients as likely having SSI if they had at least one independent variable that were included in the final models, and unlikely having SSI if they did not have any of these variables.

In the training dataset, overall 42 patients who underwent abdominal hysterectomy were classified as likely having SSI; and 277 as unlikely having SSI, thus yielding a sensitivity of 98% (95% CI 88–100), a specificity of 58% (95% CI 53–62), PPV of 17% (95% CI 12–21), and NPV of 100% (95% CI 98–100). The corresponding numbers among patients who underwent an orthopedic surgery were 52 and 848, yielding a sensitivity and specificity of 91% (95% CI 81–96) and 82% (95% CI 80–84), respectively, and PPV and NPV of 22% (95% CI 17–28) and 99% (95% CI 99–100), respectively. Among all patients who underwent colorectal surgery, there were 204 and 350 who were classified as likely or unlikely having SSI, thus yielding a sensitivity and specificity of 96% (95% CI 92–98) and 62% (95% CI 58–66), and PPV and NPV of 53% (95% CI 49–58) and 97% (95% CI 94–98), respectively (Table 4).

**TABLE 4:** Validity indices of the screening tool of SSI by surgery category–training dataset^a^.

	Screening tool (having any of the following variables based on the final logistic regression models)^b^	No. likely SSI by screening/total no. SSI	No. unlikely SSI by screening/total no. SSI	Sensitivity (95% CI)	Specificity (95% CI)	PPV (95% CI)	NPV (95% CI)
Abdominal hysterectomy, *N* = 523	Prolonged hospitalization, wound culture was ordered, blood culture was ordered, emergency room visit, reoperation	42/43	277/480	98 (88–100)	58 (53–62)	17 (12–21)	100 (98–100)
Orthopedic surgery,^c^ *N* = 1,090	Emergency room visit, wound culture was ordered, reoperation, SSI diagnosis by attending physician	52/57	848/1,033	91 (81–96)	82 (80–84)	22 (17–28)	99 (99–100)
Colorectal surgeries, all patient, *N* = 817	Emergency room visit, LOS 8 days, readmission, blood culture was ordered, wound culture was ordered	244/255	350/562	96 (92–98)	62 (58–66)	53 (49–58)	97 (94–98)
Elective colorectal surgery, *N* = 531	Readmission, wound culture was ordered, prolonged hospitalization	123/131	281/400	94 (88–97)	70 (66–74)	51 (44–57)	97 (94–98)
Urgent colorectal surgery, *N* = 286	Prolonged hospitalization, wound culture was ordered, blood culture was ordered, reoperation, emergency room visit	120/124	82/162	97 (92–99)	51 (43–58)	60 (53–66)	95 (88–98)
Colorectal surgeries-clean contaminated wound class, *N* = 645	Wound culture was ordered, prolonged hospitalization, emergency room, visit, readmission	161/173	317/472	93 (88–96)	67 (63–71)	51 (47–54)	96 (94–98)

^a^The screening tool was built based on logistic regression models, in which we identified the correlates of SSI, specifically for each surgery category. Patients who had any of the variables that were included in the final model were classified as having SSI, and those who did not have any of the variables were classified as unlikely having SSI. The gold standard was SSI as determined by an infection control physician.

^b^Prolonged hospitalization was defined as a length of stay of more than 4 days, for women who underwent abdominal hysterectomy and more than 8 days for patients who underwent colorectal surgery. These cutoffs were determined using the 75th percentiles of the length of stay among patients who did not have SSI.

^c^Orthopedic surgeries included joint replacement and spinal fusion.

CI, confidence interval; LOS, length of stay; NNP, negative predictive value; PPV, positive predictive value; SSI, surgical site infection.

The results of the correlates of SSI in the validation dataset were overall comparable to the results in the training dataset (Supplementary Tables 1, 2). Applying the screening tool to the validation dataset showed sensitivities of 89% (95% CI 67–97) and 85% (95% CI 72–93) for identifying SSI in patients who underwent abdominal hysterectomy and orthopedic surgeries, respectively, and 98% (95% CI 93–99) in those who underwent s colorectal surgery, compared to SSI diagnosis by an infection control practitioner. The specificity was 75% (95% CI 69–80), 83% (95% CI 80–86), and 59% (95% CI 53–64), respectively. PPV was 19% (95% CI 15–24) in abdominal hysterectomy, 26% (95% CI 22–31) in orthopedic surgeries and 45% (95% CI 42–48) in colorectal surgeries and NPV were 99% (95% CI 97–100) both in abdominal hysterectomy and orthopedic surgeries, and 99% (95% CI 96–100) in colorectal surgeries (Table 5).

**TABLE 5:** Validity indices of the screening tool of SSI by surgery category–validation dataset^a^.

	Screening tool (having any of the following variables)^b^	Number of likely SSI by screening/total with SSI	Number of unlikely SSI by screening/total without SSI	Sensitivity (%) (95% CI)	Specificity (%) (95% CI)	PPV (%) (95% CI)	NPV (%) (95% CI)
Abdominal hysterectomy, *N* = 289	Prolonged hospitalization, wound culture was ordered, blood culture was ordered, emergency room visit, reoperation	16/18	203/271	89 (67–97)	75 (69–80)	19 (15–24)	99 (97–100)
Orthopedic surgery,^c^ *N* = 609	Emergency room visit, wound culture was ordered, reoperation, SSI diagnosis by attending physician	35/41	471/568	85 (72–93)	83 (80–86)	26 (22–31)	99 (97–99)
Colorectal surgeries, all patients, *N* = 412	Emergency room visit, LOS 8 days, readmission, blood culture was ordered, wound culture was ordered	103/105	180/307	98 (93–99)	59 (53–64)	45 (42–48)	99 (96–100)
Elective colorectal surgery, *N* = 269	Readmission, wound culture was ordered, prolonged hospitalization	47/48	163/221	98 (89–100)	74 (68–80)	45 (40–51)	99 (97–100)
Urgent colorectal surgery, *N* = 143	Prolonged hospitalization, wound culture was ordered, blood culture was ordered, reoperation, emergency room visit	53/57	44/86	93 (83–97)	51 (41–61)	74 (69–78)	83 (66–92)
Colorectal surgeries-clean contaminated wound class, *N* = 303	Wound culture was ordered, prolonged hospitalization, emergency room, visit, readmission	62/62	159/241	100 (94–100)	66 (60–72)	43 (38–47)	100 (97–100)

^a^The screening tool was built based on logistic regression models, in which we identified the correlates of SSI, specifically for each surgery category. Patients who had any of the variables that were included in the final model were classified as having SSI, and those who did not have any of the variables were classified as unlikely having SSI. The gold standard was SSI as determined by an infection control physician.

^b^Prolonged hospitalization was defined as a length of stay of more than 4 days, for women who underwent abdominal hysterectomy and more than 8 days for patients who underwent colorectal surgery. These cutoffs were determined using the 75th percentiles of the length of stay among patients who did not have SSI.

^c^Orthopedic surgeries included joint replacement and spinal fusion.

CI, confidence interval; LOS, length of stay; NNP, negative predictive value; PPV, positive predictive value; SSI, surgical site infection.

Applying the screening tool in all surgeries in real life would have saved the full medical chart review of 1492 (61.3%) records in the training dataset and 864 (66.0%) in the validation dataset. Assuming that an infection control practitioner spends on average 10 min reviewing each patient chart, it is estimated that our screening tool has the potential of saving 248.7 and 144 working hours, respectively.

Overall, 26 SSIs of all 3,740 surgeries were missed by applying the screening tool on both the training and validation datasets. Only two of them were deep or organ-space infections.

## Discussion

We developed and validated a screening tool for the surveillance of SSIs in adults who underwent abdominal hysterectomy, colorectal surgeries, and orthopedic surgeries (i.e., spinal fusion and joint replacements). The screening tool comprised simple demographic and clinical characteristics of the patient, surgery and management. The main findings are that the screening tool displayed good validity indices, in both the training and validation datasets and can be easily integrated in semi-automated SSI surveillance systems. Moreover, the constructed screening tool showed promising potential of saving in workload, thus reducing the burden on the infection control units that results from manual chart review in the framework of SSI surveillance. Applying the screening tool to both the training and validation datasets suggested a reduction of about 60% in the number of medical records that need to be fully reviewed manually, while accurately detecting nearly all patients with SSI.

In practice, patient charts that will have one or more of the variables mentioned above will be flagged for the infection control practitioner, a summary of flagged charts can be prepared periodically. Infection control teams will review only flagged charts thus saving considerable time and workload. These activities will prompt an expedited verification and identification process of SSI in these patients. This is expected to enhance the feedback given to surgeons in the framework of SSI surveillance program, and reduce the burden associated with SSI more efficiently.

The ability of our screening tool and other similar tools (17, 19, 20, 26, 27) to accurately exclude a large amount of records from review is expected to improve the quality and extend the surveillance of SSI, which is compromised in many medical facilities due to shortage in infection control personnel and limited resources.

We found some differences across surgeries in the correlates of SSI and within colorectal surgeries, there might be also some differences between sub-categories (e.g., urgent, vs. elective). These findings suggest that surgery specific characteristics should be employed when developing screening tools for SSI. The correlates of SSI in the training dataset were similar to those found in the validation dataset, although some were not statistically significant in the latter, likely due to the smaller sample size. Despite these differences between the training the validation datasets, the screening tool was highly sensitive when it was applied on the validation dataset.

The documentation of SSI diagnosis in the medical records was low in all surgery categories thus enforcing the essential need of SSI designated surveillance system as a basis for the detection and planning prevention and control interventions.

Similar to our study, most studies on surveillance algorithms focused on one or few surgery categories. In a study from Korea focused on multiple surgeries (20) an algorithm was developed based on 3 characteristics to survey 38 surgery categories reaching high sensitivity of 96.7%: antibiotic prescribing, ordering microbiological studies and ordering consultation with an infectious disease specialist. Worth mentioning is that ordering consultation with infectious disease specialist might depend on local practices and resources, thus might not be generalizable to other settings and populations. Antibiotic treatment and microbiology tests were found to be strongly associated with SSI in other studies (28, 29), nonetheless the duration of antibiotic treatment might vary across surgeries. For instance, in a multicenter study on coronary artery bypass graft (CABG) a ≥ 9 days of antibiotic treatment had excellent prediction of SSI, while a study among women who underwent cesarean section found a threshold of ≥ 2 days of antibiotic treatment predicted SSI (28, 29).

We did not include antibiotic prescribing in the present model because our administrative data do not accurately capture this information. Our model may have performed better with accurate access to pharmacy data but even so we managed to achieve high detection rate with the available administrative, demographic and clinical data. Nevertheless, information systems in hospitals are improving constantly and information regarding antibiotic prescription or other variables can integrate into the prediction model in the future.

Surveillance of SSI in some hospitals uses positive microbiologic cultures for the detection of SSI (17). Being very specific, looking only for positive cultures might miss SSIs in patients with negative cultures or when other criteria such as radiologic evidence of infection or physician’s diagnosis, without positive culture, are fulfilled. Accordingly, we used in our screening tool the wider criteria of ordering microbiological culture and did not restrict it to only positive cultures, thus allowing for capturing nearly all SSIs in our datasets. Conversely, the specificity was lower than the sensitivity suggesting the potential for even further reduction in the number of medical records that need to be fully reviewed. From SSI surveillance perspective, which should be followed by a feedback to surgeons to improve patient’s outcomes and reduce the burden associated with SSI, we believe that a higher sensitivity is important over the specificity.

Considering ours and others’ findings (28, 29), the effort to create one prediction model or screenaing tool for all surgery categories seems to be less feasible and might be less desired taking into account the differences in patient characteristics, hospitalization practices and infection nature. Nonetheless, we showed shared correlates of SSI across surgeries and the two datasets. Collectively these findings suggest the existence of stable core characteristics that might be broadly used in SSI surveillance, although periodical testing or verification of the screening tool is likely warranted.

The ability to capture post discharge SSI became more important once LOS after surgery is significantly shortened (30–33) and a larger fraction of SSIs is diagnosed post-discharge. Clinically significant SSIs often require surgical intervention such as wound reopening, surgical debridement or drainage and sometimes even major operation and antibiotic treatment. Methods to identify post-discharge SSIs include direct observation of surgical wound, telephone interviews with patients or surgeons, capturing readmissions or surgical revisions, review of pharmacy data and mixed methods (14). In our study, emergency room visits and readmissions were included to capture post-discharge SSIs.

In the last years, benchmark of hospital-acquired infections, for comparison between hospitals become an important focus. Using both standardized infection definitions and standardized detecting methods is essential to allow accurate comparisons across hospitals. In SSI surveillance and surveillance as a rule, there is a great impact of the method used on case finding (12). When applying detection algorithms, it is important to create a uniform algorithm containing variables that are easy to capture by all facilities being compared (27).

Fully automated surveillance, using machine learning, Natural Language Processing (NLP) or Bayesian network will expanse further the ability to survey wide range of surgeries and save even more time and work load but it will take a while until it will be routinely used (34–36). The utilization of screening tools of SSI as described in our study will fill such an important gap.

Of note, classic risk factors such as high NNIS risk index were significantly correlated with SSI in orthopedic and colorectal surgeries but were not significant in in the final multivariable logistic regression. This might suggest that these factors are important for risk stratification but not suitable as correlates for SSI diagnosis.

Our study has some limitations, first, it is a single-center study, which might limit the generalizability of our findings. Nonetheless, the fact that this is a single center study might be considered a strength given the relatively homogenous practice. Moreover, the variables that were used in building the models to identify SSI are generic and are easily found in other hospitals of health systems. Second, there significant differences in some of the patients’ and surgeries’ characteristics between study periods. However, a close examination of these differences showed that although they were statistically significant they were mostly of small magnitude. Realizing that part of those changes are inevitable demonstrates the need for constant evaluation of HAI and SSI detection algorithms. Third, due to the COVID-19 pandemic in 2020, the number of elective surgeries was smaller than we expected in the validation period. Fourth, some SSIs that might be diagnosed and managed in the community or in other hospitals were probably missed. However, we assume that patients with clinically significant SSI will likely be referred or return to their operating surgeon and hospital more than other hospitals. Accordingly, we believe that we captured the majority of clinically significant SSIs. Our screening stool, utilizing simple variables is suitable for routine surveillance of SSI and can also be applied by other institutions.

Strengths of our study include using data spanning over a 6-year period with large sample sizes, multiple surgeries categories, the diagnosis of SSI, which was determined by trained infection control specialists using standardized accepted criteria, and the availability of a wide range of patients and surgery characteristics.

Of note, infection control practices such as improving antimicrobial prophylaxis, skin antisepsis and sterile technique implemented as necessary across both study periods. The division into test and validation datasets was arbitrary and did not necessarily correspond to those interventions.

## Conclusion

We demonstrated the development of semi-automated screening tool for SSI surveillance, which had high sensitivity and fair specificity. The tool is based on simple demographic and clinical characteristics of patients and surgeries that can be easily extracted from patients EMRs. The integration of such a screening tool in the surveillance process will allow infection control team to review only flagged patient files, and has a great potential of reducing workload, saving time and improving the surveillance of SSI.

## Data availability statement

The raw data supporting the conclusions of this article will be made available by the authors, without undue reservation.

## Ethics statement

The studies involving human participants were reviewed and approved by Institutional Review Board Committee of Meir Medical Center. Written informed consent for participation was not required for this study in accordance with the national legislation and the institutional requirements.

## Author contributions

PS and KM designed the study. PS drafted the initial manuscript. KM and MC revised the manuscript critically for important intellectual content. All authors participated in the acquisition, analysis, and interpretation of the data, read, and agreed to the published version of the manuscript.
