# Supplementary material for: The development and validation of screening tools for semi-automated surveillance of surgical site infection following various surgeries
Source: Front Med (Lausanne). 2023 Jan 26;10:1023385. doi: 10.3389/fmed.2023.1023385 (PMC9909272; doi:10.3389/fmed.2023.1023385)
Supplement: Supplementary file 1 [file Table_1.docx]

**Supplementary Table 1: Factors associated with surgical site infection by surgery category; bivariate analysis by surgery category- validation dataset**

|  | **Abdominal hysterectomy (N=289)** | | | **Orthopedic surgery (N=609)** | | | **Colorectal surgery (N=412)** | | |
| --- | --- | --- | --- | --- | --- | --- | --- | --- | --- |
|  | **No SSI (N=271)** | **SSI (N=18)** | **P** | **No SSI (N= 568)** | **SSI (N= 41)** | **P** | **No SSI (N=307)** | **SSI (N=105)** | **P** |
| Age, years median (IQR) | 55.0 (47.0-68.0) | 64.0 (43.0-72.5) | 0.8 | 68.5 (59.3-75.8) | 66 (60.5- 74.0) | 0.7 | 67.0 (53.0-78.0) | 68.0 (55.0-77.5) | 0.74 |
| Sex, male, N (%) | N/A | N/A |  | 275 (48.4) | 10 (24.4) | 0.8 | 157 (51.0) | 51 (49.0) | 0.65 |
| Anesthesia (general), N (%) | 268 (99.0) | 17 (94.4) | 0.2 | 441 (77.6) | 33 (81.0) | 0.7 | 306 (99.7) | 104 (99) | 0.45 |
| Urgent surgery, N (%) | 2 (0.7) | 2 (11.0) | <0.001 | 153 (27.0) | 18 (44) | 0.02 | 86 (28.0) | 57 (54.3) | <0.001 |
| Received antibiotic prophylaxis, N (%) | 265 (98) | 18 (100) | 0.5 | 551 (97.0) | 41 (100) | 0.62 | 288 (94.0) | 95 (90.0) | 0.14 |
| Emergency room visit, N (%) | 33 (12.2) | 11 (61) | <0.001 | 61 (10.7) | 25 (61.0) | <0.001 | 36 (12.0) | 23 (22.0) | 0.01 |
| Readmission, N (%) | 27 (10) | 10 (56.0) | <0.001 | 76 (13.4) | 29 (71.0) | <0.001 | 53 (17.3) | 40 (38.0) | <0.001 |
| Blood culture ordered, N (%) | 17 (6.3) | 10 (56.0) | <0.001 | 49 (8.6) | 20 (49) | <0.001 | 43 (14.0) | 51 (49.0) | <0.001 |
| Wound culture ordered, N (%) | 6 (2.2) | 10 (56.0) | <0.001 | 20 (3.5) | 23 (56) | <0.001 | 22 (7.2) | 58 (55.2) | <0.001 |
| Reoperation, N (%) | 10 (3.7) | 4 (22.2) | 0.007 | 37 (6.5) | 27 (66) | <0.001 | 60 (19.5) | 49 (47.0) | <0.001 |
| Documentation of SSI diagnosis in the medical chart, N (%) | 0 (0.0) | 3 (16.7) | <0.001 | 6 (1.1) | 19 (46.3) | <0.001 | 1 (0.3) | 10 (9.5) | <0.001 |
| High-grade ASA (>3), N (%) | 82 (30.0) | 8 (44.4) | 0.2 | 249 (44) | 21 (51) | 0.36 | 137 (44.6) | 55 (52.4) | 0.17 |
| High-risk index (2-3), N (%) | 52 (19.2) | 6 (33.3) | 0.2 | 72 (13.0) | 10 (24.4) | 0.034 | 84 (27.4) | 47 (44.8) | <0.001 |
| High-grade wound class (contaminated, dirty), N (%) | 3 (1) | 1 (5.6) | 0.23 | 32 (5.6) | 6 (14.6) | 0.035 | 66 (21.5) | 43 (41.0) | <0.001 |
| Diabetes, N (%) | 10 (3.7) | 2 (11.0) | 0.17 | 88 (15.5) | 6 (14.6) | 0.9 | 41 (13.4) | 15 (14.3) | 0.8 |
| Operation duration ,hours, median (IQR) | 2.36 (2-3.2) | 2.35 (2.1-3.1) | 0.5 | 1.6 (1.4-2.3) | 2.0 (1.4-2.6) | 0.23 | 2.5 (1.6-3.5) | 2.5 (2.2-3.5) | 0.5 |
| LOS before operation, days, median (IQR) | 1 (1-1) | 1 (1-1) | 0.65 | 1 (1-1) | 1 (1-3) | 0.009 | 1 (1-1) | 1 (1-2) | 0.8 |
| LOS from surgery to discharge, days, median (IQR) | 2 (2-4) | 3 (2-9.5) | 0.05 | 4 (3-7) | 5 (3-13.5) | 0.04 | 5 (4-8) | 12 (8-21) | <0.001 |
| Prolong hospitalization ^a^, N (%) | 33 (22) | 7 (39) | 0.006 | 123 (22.0) | 17 (41.5) | 0.004 | 67 (22.0) | 75 (71.4) | <0.001 |

^a^ Prolonged hospitalization was defined as a length of stay of more than 4 days for women who underwent an abdominal hysterectomy, more than 6 days for orthopedic surgeries, and more than 8 days for patients who underwent colorectal surgery. These cutoffs were determined using the 75th percentiles of the length of stay among patients who did not have SSI.

ASA: American Society of Anesthesiologists IQR: interquartile range; LOS: length of stay; SSI: surgical site infection;

P value was obtained by the Student’s *t* test for continuous variables or the Mann-Whitney test for variables that did not follow a normal distribution, and the chi square or Fisher exact where appropriate for categorical variables.
