# Supplementary material for: The development and validation of screening tools for semi-automated surveillance of surgical site infection following various surgeries
Source: Front Med (Lausanne). 2023 Jan 26;10:1023385. doi: 10.3389/fmed.2023.1023385 (PMC9909272; doi:10.3389/fmed.2023.1023385)
Supplement: Supplementary file 2 [file Table_2.DOCX]

Supplementary Table 2: Multivariable logistic regression models of factors associated with the development of SSI by surgery category – validation dataset

| Surgery category | Variable | Adjusted OR | 95% CI | P value | Negelkerk R square | AUC  (95% CI) ^c^ |
| --- | --- | --- | --- | --- | --- | --- |
| Abdominal hysterectomy |  |  |  |  | 0.48 | 0.90  (0.80-0.99) |
|  | LOS>4 days (vs. 4 days or less) | 1.2 | 0.2-6 | 0.9 |  |  |
|  | Emergency room visit (yes vs. no) | 2.7 | 0.6-11.3 | 0.2 |  |  |
|  | Wound culture ordered (yes vs. no) | 23.4 | 5.0-109 | <0.001 |  |  |
|  | Blood culture ordered (yes vs. no) | 8.5 | 1.8-39 | 0.006 |  |  |
|  | Reoperation (yes vs. no) | 1.6 | 0.2-10.5 | 0.6 |  |  |
| Orthopedic surgery |  |  |  |  | 0.55 | 0.90  (0.83-0.97) |
|  | Emergency room visit (yes vs. no) | 1.9 | 0.7-5.2 | 0.2 |  |  |
|  | Wound culture ordered (yes vs. no) | 8.4 | 3.0-24.0 | <0.001 |  |  |
|  | Reoperation (yes vs. no) | 7.6 | 2.9-20.0 | <0.001 |  |  |
|  | Documentation of SSI diagnosis in the medical chart (yes vs. no) | 22.3 | 6.3-79.0 | <0.001 |  |  |
| Colorectal surgery, all patients |  |  |  |  | 0.50 | 0.90  (0.86-0.93) |
|  | Emergency room visit (yes vs. no) | 2.0 | 0.9-4.6 | 0.1 |  |  |
|  | Wound culture ordered (yes vs. no) | 11.6 | 6.0-22.5 | <0.001 |  |  |
|  | Blood culture ordered (yes vs. no) | 3.6 | 1.9-6.8 | <0.001 |  |  |
|  | Readmission (yes vs. no) | 1.9 | 0.96-3.9 | 0.065 |  |  |
|  | LOS>8 days (vs. 8 days or less) | 4.4 | 2.3-8.3 | <0.001 |  |  |
| Elective colorectal surgery |  |  |  |  | 0.59 | 0.93  (0.88-0.97) |
|  | Readmission (yes vs. no) | 6.4 | 2.4-16.9 | <0.001 |  |  |
|  | LOS>8 days (vs. 8 days or less) | 4.9 | 1.9-12.4 | <0.001 |  |  |
|  | Wound culture ordered (yes vs. no) | 50.2 | 16.3-154.6 | <0.001 |  |  |
| Urgent colorectal surgery |  |  |  |  | 0.28 | 0.78  (0.70-0.86) |
|  | Emergency room visit (yes vs. no) | 1.4 | 0.4-4.4 | 0.6 |  |  |
|  | Wound culture ordered (yes vs. no) | 3.2 | 1.4-7.4 | 0.006 |  |  |
|  | Blood culture ordered (yes vs. no) | 1.6 | 0.7-3.9 | 0.3 |  |  |
|  | Reoperation (yes vs. no) | 1.1 | 0.5-2.6 | 0.8 |  |  |
|  | LOS>8 days (vs. 8 days or less) | 4.1 | 1.6-10.4 | 0.003 |  |  |
| Clean-contaminated colorectal surgery |  |  |  |  | 0.55 | 0.93  (0.90-0.96) |
|  | Wound culture ordered (yes vs. no) | 21.9 | 8.8-54.3 | <0.001 |  |  |
|  | LOS>8 days (vs. 8 days or less) | 7.7 | 3.5-17.2 | <0.001 |  |  |
|  | Emergency room visit (yes vs. no) | 2.6 | 0.9-7.3 | 0.07 |  |  |
|  | Readmission (yes vs. no) | 2.5 | 1.0-6.3 | 0.046 |  |  |

^a^ AUC: area under the curve; CI: confidence intervals; LOS: length of stay; OR: odds ratio; SSI: surgical site infection;

Prolonged hospitalization was defined as a length of stay of more than 4 days, for women who underwent abdominal hysterectomy and more than 8 days for patients who underwent colorectal surgery. These cutoffs were determined using the 75th percentile of the length of stay among patients who did not have SSI.

^b^ P value by Hosmer and Lemeshow was 0.57, 0.73, <0.001 for abdominal hysterectomy, orthopedic surgeries and colorectal surgeries (all patients). The respective P values for elective colorectal surgeries, clean contaminated wound class, and urgent colorectal surgery were 0.016, 0.017, and 0.07.

^c^ Area under the curve of the entire model.
